# Sealing Performance of Phenyl-Silicone Rubber Based on Constitutive Model Under Thermo-Oxidative Aging

**DOI:** 10.3390/polym18030350

**Published:** 2026-01-28

**Authors:** Haiqiang Shi, Jian Wu, Zhihao Chen, Pengtao Cao, Tianxiao Zhou, Benlong Su, Youshan Wang

**Affiliations:** 1 Center for Rubber Composite Materials and Structures, Harbin Institute of Technology, Weihai 264209, China; zheshigexianjing@163.com (H.S.); 22b908093@stu.hit.edu.cn (Z.C.); cptcsu@163.com (P.C.); 17828025346@163.com (T.Z.); subenlong@hit.edu.cn (B.S.); wangys@hit.edu.cn (Y.W.); 2 National Key Laboratory of Science and Technology on Advanced Composites in Special Environments, Harbin Institute of Technology, Harbin 150090, China

**Keywords:** thermo-oxidative aging, lifetime prediction, Neo-Hookean hyperelastic model, Omega-profile seal

## Abstract

Phenyl-silicone rubber is the elastomer of choice for cryogenic and high-temperature static seals, yet quantitative links between thermo-oxidative aging and sealing reliability are still lacking. Here, sub-ambient (−70 °C to 25 °C) and room-temperature mechanical tests, compression set aging, SEM, FT-IR, and finite-element simulations are integrated to trace how aging translates into contact-pressure decay of an Omega-profile gasket. Compression set rises monotonically with time and temperature; an Arrhenius model derived from 80 to 140 °C data predicts 34 d (10% set) and 286 d (45% set) of storage life at 25 °C. SEM reveals a progressive shift from ductile dimple fracture to brittle, honeycomb porosity, while FT-IR confirms limited surface oxidation without bulk chain scission. Finite element analyses show that contact pressure always peaks at the two lateral necks; short-term aging increases in the shear modulus C_10_ from 1.87 to 2.27 MPa, raising CPRESS by 8~21%, yet this benefit is ultimately offset by displacement loss from compression set (8.0 mm to 6.1 mm), yielding a net pressure reduction of 0.006 MPa. Critically, even under the most severe coupled condition (56 days aging with compression set), the predicted CPRESS remains above the 0.1 MPa leak-tightness criterion across the entire cryogenic service envelope. This framework provides deterministic boundaries for temperature, aging duration, and allowable preload relaxation, enabling risk-informed maintenance and replacement scheduling for safety-critical phenyl-silicone seals.

## 1. Introduction

Phenyl-silicone rubber has become the elastomer of choice for safety-critical seals in aerospace propulsion, high-end electromechanics, automotive powertrains and marine transmissions because it couples wide-temperature flexibility with chemical inertness, electrical insulation, wear, weather and ozone resistance; nevertheless, the same aggressive environments subject it to a complex thermos-oxidative aging environment that progressively densifies the network, raises hardness and compression set, roughens the surface and ultimately compromises static sealing. Clarifying how thermos-oxidative aging proceeds and how it degrades sealing reliability between −70 °C and 25 °C is therefore an urgent prerequisite for integrated material-structure-performance design.

The literature offers an extended genealogy: Rey [1] first identified temperature as the master variable governing the mechanical response of silicone rubber, while Shimada and Hanada [2,3] quantified hardness degradation and coupled property variations in cable-grade rubbers exposed to simultaneous heat, oxygen, and radiation; Wu [4] used XPS and NMR to show that thermo-oxidative aging increases hardness, compression set, and surface roughness in lock-step with the loss of seal ability; Kashi and Ito [5,6] mapped the mechano-chemical pathways that dominate in hot oil and demonstrated that the cross-link architecture dictates aging properties, proposing rapid early-stage diagnostics; Huang [7] polymerized methylphenyl and diphenyl cyclo-siloxanes and showed that pendant phenyl groups, especially in diphenyl sequences, markedly improve thermal stability, with DMTA revealing a 4.1 MPa tensile-strength gain at −70 °C; Englert, Pires, Han, He, and Wang [8,9,10,11,12] subsequently, verified the synergistic benefit of TiO_2_ fillers, elucidated the degradation chemistry of fluoro-silicone rubbers, and demonstrated that CeO_2_/graphene or porous calcium silicate-supported CeO_2_ further retard oxidation. Wang [13] conducts voltage withstand tests on abnormal heating composite insulators under varying humidity, analyzes temperature characteristics via infrared and ultraviolet imaging, and measures micro-morphology, chemical groups, and dielectric properties, finding that material deterioration causes abnormal heating exacerbated by high humidity. Chen [14] found that accelerated aging of RTV anti-pollution coatings induces cross-linking/oxidative scission, which deteriorates bulk and surface properties as quantified by color, gloss, topography, wettability, and insulation loss. Li [15] systemically investigated the oil-thermal aging of nitrile-butadiene rubber seals by experimentally tracking the evolution of elastic modulus, compression-set, friction coefficient, contact pressure, and effective sealing area and embedding the derived aging coefficients into a coupled finite-element model that mechanistically links oxidative degradation to the long-term sealing reliability. Beyond the elastomer material, Masson [16] correlated surface roughening, stiffness loss, and silanol formation during 30 days immersion in pH 13.5 alkali and predicted >1 year shelf life; Porter [17] accelerated nitrile O-rings at 40~85 °C and found specification compliance for 7 years, followed by irreversible elasticity loss; Goudarzi [18] modeled insulator aging through the carbonyl index with ≤±6% residual-life error; Taourit [19] coupled chemiluminescence oxidation kinetics with fatigue data to define the embrittlement limit of unfilled natural rubber after the oxidation induction time; Dong and Dong [20,21] quantified the anisotropic stiffness of woven rubber laminates from −40 °C to 200 °C and showed that a ±55° bias-ply layout extends seal life by 30%; Xu [22] developed a non-orthogonal mesoscopic model that captures the crimp-lock mechanism of 3-D braided reinforcements and enables seals to retain 85% of their room-temperature burst pressure at 180 °C; Jin [23] formulated a temperature-augmented Mooney−Rivlin model that keeps the HNBR/FKM contact pressure above the formation pressure at 200 °C; Zhang [24] closed the loop from material to application by lining helicopter landing-gear dynamic seals with PTFE composites that cut wear by 55% after 5000 cycles.

The innovations of this study are threefold: establishing a comprehensive framework for understanding and predicting phenyl-silicone rubber seal degradation. First, we develop a multi-scale coupled analysis framework that transcends the limitations of existing studies by synchronously integrating mechanical testing across a broad cryogenic-to-ambient temperature range (−70 °C to 25 °C), compression set experiments, SEM microstructure characterization, and FT-IR chemical analysis. This creates an unprecedented mechanistic linkage between thermo-oxidative aging-induced chemical changes, microstructural damage evolution, and macroscopic sealing performance decay, thereby elucidating the fundamental origins of seal reliability loss. Second, we systematically characterize aging behavior across a wide temperature domain through the first comprehensive thermo-oxidative aging experiments spanning cryogenic to ambient conditions, thereby filling a critical research gap in understanding silicone rubber degradation in extreme cryogenic environments and providing essential empirical data to support the design and validation of aerospace and other low-temperature sealing applications. Third, we construct and validate an aging-aware constitutive model by embedding temperature- and time-dependent mechanical parameters into a Neo−Hookean hyperelastic framework while simultaneously incorporating displacement loss induced by compression set. This integrated modeling approach enables accurate prediction of contact pressure distributions in Omega-profile seals following long-term thermo-oxidative exposure, thereby providing a quantitative, physics-based tool for reliability assessment and maintenance scheduling of critical sealing components.

## 2. Materials and Methods

### 2.1. Materials

The silicone rubber employed in this study is a phenyl-modified grade supplied by the Beijing Institute of Aeronautical Materials, China.

### 2.2. Sample Preparation

Dumbbell samples were prepared strictly in accordance with ISO 23529:2016 [25] and ISO 37:2017 [26]. Type-2 dumbbells were machined from the phenyl-silicone rubber sheet; their nominal dimensions are listed in Table 1.

Cylindrical samples for compression set tests were produced following ISO 815-1:2014 [27]. Each cylinder measures 10.0 ± 0.2 mm in height and 10.0 ± 0.2 mm in diameter.

### 2.3. Thermo-Oxidative Accelerated Aging Tests

Aging was performed in a 401 A air-circulating oven (Daochun Test Machinery, China) whose temperature range is 25~300 °C with a control accuracy of ±0.5 °C. The procedure was strictly followed according to ISO 188:2011 [28].

To simulate service conditions, aging temperatures were set at 80 °C, 100 °C, 120 °C, and 140 °C. Dumbbell samples were removed at the intervals specified in Table 2 for uniaxial tensile tests (ISO 37:2017); cylinders were taken out for compression set evaluation (ISO 815-1:2014). Three replicate samples were used at each condition.

### 2.4. Characterization

#### 2.4.1. Mechanical Properties

Hardness Testing: An LX-A Shore A durometer was used on the cylindrical samples according to ISO 7619-2:2010 [29]. Five measurements were taken on each sample before and after aging; the average was used.

Uniaxial Tensile Testing: Performed on a high−low temperature universal testing machine at 500 mm/min with ±0.01 mm displacement accuracy and a video extensometer for real-time strain monitoring. Tensile strength and elongation at break were recorded; results are the mean of three samples.

An environmental chamber (Table 3) equilibrated samples at the required temperature for 10 min prior to testing.

#### 2.4.2. Compression Set

Compression set tests were carried out according to ISO 815-1:2014. Cylindrical samples (Φ10 × 10 mm) were compressed to 30% deflection using a steel fixture (110 × 50 × 10 mm, Ra ≤ 0.4 µm) and a 7 ± 0.02 mm spacer. After the prescribed aging period, samples were unloaded and allowed to recover for 30 min in a standard atmosphere. Recovery height was measured with a 0.01 mm precision micrometer, and the compression-set ratio K was calculated as

(1) $$K=\frac{\left(h_{0}-h_{2}\right)}{\left(h_{0}-h_{1}\right)}\;\times \;100\%$$

where $h_{0}$, $h_{2}$, and $h_{1}$ are the original, recovered, and spacer heights, respectively. Results are the average of three samples.

#### 2.4.3. SEM

Surface morphology before and after aging was examined with a Zeiss MERLIN Compact field-emission SEM at high resolution to reveal nano-scale cracking and topography variations.

#### 2.4.4. FT-IR

A Bruker Tensor II spectrometer was employed (4000~400 cm^−1^, 4 cm^−1^ resolution, 32 scans). Spectra were recorded in absorbance mode; changes in characteristic peak intensity and the appearance of new bands were used to quantify oxidation and degradation products.

### 2.5. Life Prediction Method

Assuming that the activation energy (Ea) of the dominant degradation chemistry remains invariant, the compression set retention Y = 1 − ε is empirically linked to aging time t through Equation (2) with α = 0.96; linear regression of ln t against Y for phenyl-silicone data at each test temperature therefore delivers ln K, the logarithm of the rate constant that corresponds to any prescribed failure threshold. Because the Arrhenius relation (3) demands that ln K scale linearly with 1/T, slope Ea/R, a second regression of ln K versus 1/T generates a master curve that translates any isothermal rate constant into the storage temperature domain; once K at storage temperature is known, the same empirical law (2) is inverted to predict the time t required to reach the target retention Y, thereby closing the lifetime calculation.

(2) $$Y=Ae^{-Kt^{\alpha }}$$

(3) $$K=Be^{-\frac{\mathrm{Ea}}{\mathrm{RT}}}$$

### 2.6. Finite Element Model of the Seal

#### 2.6.1. Neo−Hookean Hyperelastic Model

A constitutive model for phenyl-silicone rubber was developed, and a two-dimensional simulation model of the Omega-profile hatch seal structure was established using Abaqus simulation software(6.12). Contact characteristics, such as average contact pressure, served as core metrics for evaluating the performance of different seal structures. The study systematically investigated the influence patterns of aging time and temperature environments on the contact characteristics of the seal structure.

The Neo−Hookean hyperelastic model with one-term expansion is expressed as

(4) $$W=\frac{C_{10}}{2}\left({\bar{I}}_{1}-3\right)+\frac{1}{D_{1}}{(J-1)}^{2}$$

where $C_{10}$ denotes the shear modulus of the rubber, a key parameter characterizing its resistance to shear deformation; ${\bar{I}}_{1}$ is the invariant of the strain tensor; and $D_{1}$ represents the incompressibility coefficient, quantifying the material’s volumetric rigidity under the assumption of near incompressibility.

Table 4 consolidates the Neo−Hookean hyperelastic parameters obtained by fitting the compressive modulus of phenyl-silicone rubber measured at −70 °C, −55 °C, −25 °C, 0 °C, and 25 °C after 0 and 7 days of aging, and further records the variations in these parameters following thermo-oxidative exposure for 0, 14, 28, 42, and 56 days.

#### 2.6.2. Boundary and Load Conditions

**Figure 1:**
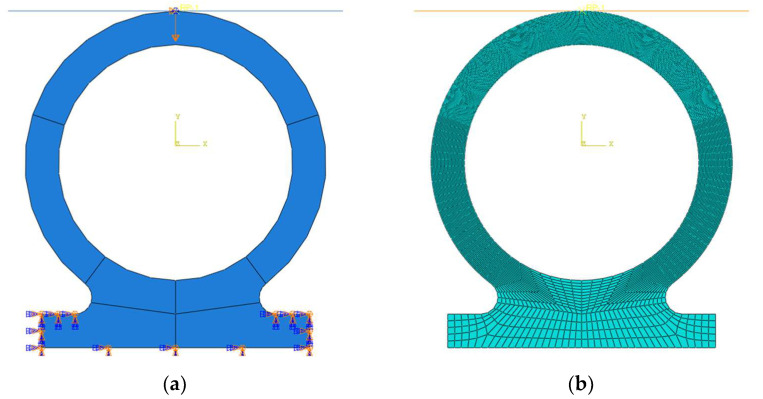
Boundary conditions and mesh generation for Omega-profile seals: (**a**) Boundary and load conditions; (**b**) mesh generations.

A two-dimensional finite element model of the Omega-profile seal structure was established using the Abaqus software (6.12), as illustrated in Figure 1a. This model is primarily composed of a metal shaft and an Omega-profile seal section. Specifically, the metal shaft was constructed as an analytical rigid body, which eliminates the need to assign material properties to it. In contrast, the Omega-profile seal structure was fabricated from phenyl silicone rubber, a material that was defined as hyperelastic in the simulation. To accurately characterize the hyperelastic and nearly incompressible properties of this rubber material, Poisson’s ratio was assigned a value of 0.495. In the load module of Abaqus, the bottom edge of the Omega-profile seal structure was fully constrained to restrict all its degrees of freedom. Subsequently, a compressive load was applied to the sealing structure by controlling the downward displacement of the metal shaft, and this loading process was continued until the maximum compressive displacement of 8 mm was achieved.

#### 2.6.3. Mesh Generations

The mesh generation of the Omega-profile seal structure was conducted within the meshing module of Abaqus. To balance the trade-off between computational accuracy and efficiency, a global mesh size of 0.5 mm was specified for the entire Omega-profile seal structure, while a refined mesh size of 0.1 mm was adopted for the contact interface region between the metal shaft and the sealing structure, where stress and deformation gradients are expected to be relatively high. The four-node bilinear plane strain hybrid quadrilateral element (CPE4RH) was selected for the numerical simulation, and the neutral axis algorithm was employed to generate tetrahedral mesh elements. Considering the hyperelastic constitutive model of the phenyl silicone rubber material, the hybrid formulation was enabled in the element type settings to effectively address the nearly incompressible mechanical behavior of the rubber, which is critical for avoiding numerical instabilities such as volumetric locking during the compression simulation. The final mesh configuration of the Omega-profile seal structure is presented in Figure 1b, consisting of a total of 8762 nodes and 9138 elements.

## 3. Results and Discussion

### 3.1. The Effect of Aging on Mechanical Properties

**Figure 2:**
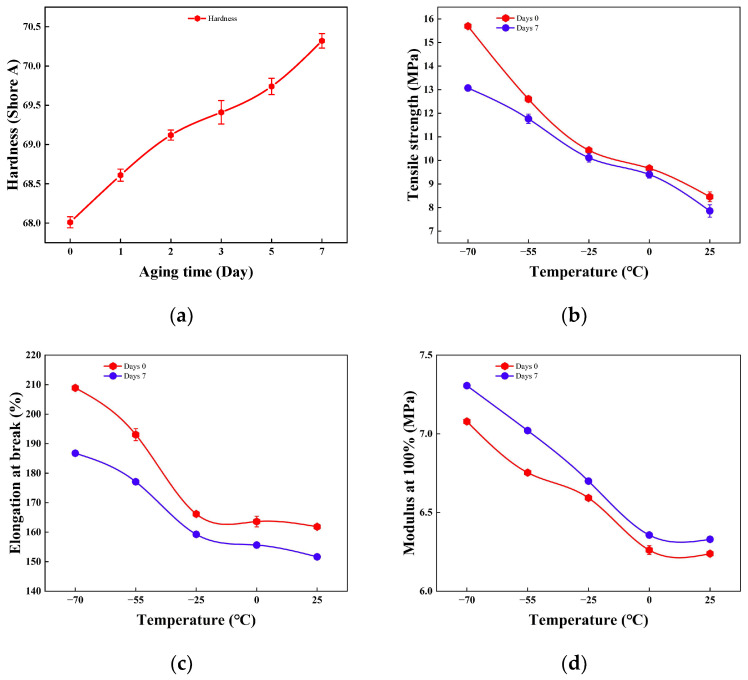
Variations in mechanical properties during thermo-oxidative aging: (**a**) hardness; (**b**) tensile strength; (**c**) elongation at break; (**d**) 100% modulus.

Figure 2 characterizes the evolution of mechanical properties for phenyl-silicone rubber following 7 days of thermo-oxidative aging. As shown in Figure 2a, Shore A hardness increases monotonically from 68.01 HA to 70.32 HA (a gain of 2.31 HA) with aging time, reflecting progressive densification and uniformity of the internal crosslink network under thermal-oxidative conditions. During temperature elevation from −70 °C to 25 °C, Figure 2b–d reveals synergistic degradation effects: aged tensile strength decreases from 13.07 MPa to 7.86 MPa, 0.3~2.6 MPa lower than unaged samples at equivalent temperatures, while aged elongation at break declines from 186% to 151%, representing an additional 7~22% loss compared to pristine material. Conversely, the 100% modulus exhibits mild temperature dependence but remains elevated by 0.1~0.3 MPa across all test temperatures relative to unaged states, confirming that thermo-oxidative aging induces network hardening while preserving the qualitative temperature sensitivity of stiffness. Collectively, these data demonstrate that short-term aging stiffens the rubber through enhanced crosslinking at the expense of ductility and ultimate strength, establishing a mechanistic foundation for subsequent sealing performance analysis.

**Figure 3:**
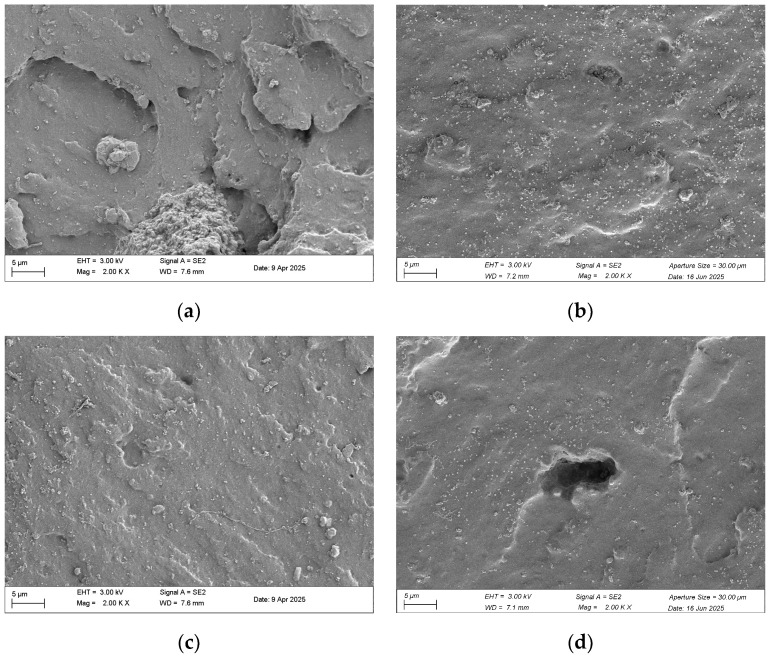
Fracture surface microstructure of phenyl-silicone rubber dumbbell samples after thermo-oxidative aging: (**a**,**b**) edge fracture morphology: unaged and 7 days aged (2000×); (**c**,**d**) interior fracture surface: unaged and 7 days aged (2000×).

Figure 3 presents a systematic SEM fractographic analysis at 2000× magnification that elucidates how seven days of thermo-oxidative aging transform the failure micro-mechanisms of phenyl-silicone dumbbell samples, thereby rationalizing the macroscopic loss of compliance and sealing reliability. In the fracture edge region (Figure 3a,b), oxidative degradation diminished step-like features and reduced overall roughness while generating numerous shallow, near-circular pits with relatively planar morphology. By contrast, the interior fracture zone (Figure 3c,d) revealed more severe damage, manifested as larger, irregular erosion cavities with coarse peripheries accompanied by outward-propagating radial microcracks.

### 3.2. The Effect of Aging on Sealing Life Prediction

**Figure 4:**
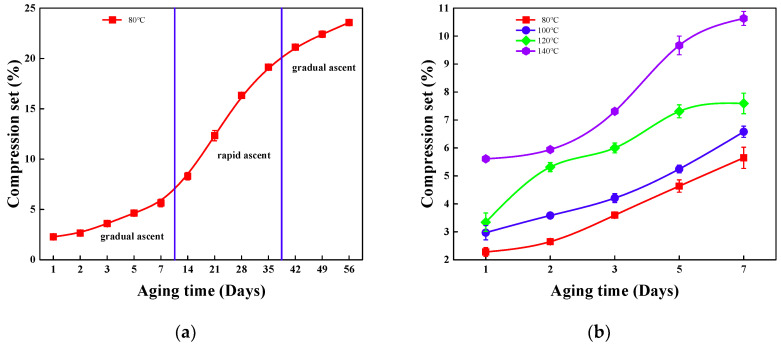
Compression set variations in phenyl-silicone rubber during thermo-oxidative aging (1, 2, 3, 5, 7, 14, 21, 28, 35, 42, 49, 56 days) at different temperatures (80, 100, 120, 140 °C): (**a**) compression set with aging time ranges; (**b**) compression set with temperature ranges.

Figure 4 illustrates the changes in the compression set of phenyl silicone rubber during thermal oxidative aging under different temperature conditions (80, 100, 120, and 140 °C) over specified aging periods (1, 2, 3, 5, 7, 14, 21, 28, 35, 42, 49, and 56 days). Specifically, Figure 4a depicts the variation in compression set with aging time, revealing how the material’s permanent deformation evolves progressively as the thermal oxidative aging process proceeds, while Figure 4b presents the relationship between compression set and aging temperature, illustrating the influence of different thermal environments on the material’s compression recovery performance after the designated aging durations. Together, the graphs demonstrate that longer aging and higher temperature act synergistically to lock additional crosslinks into the network, thereby diminishing the fraction of recoverable deformation.

At 80 °C, the compression-set curve first traverses a protracted, gradual ascent stage, during which the rate rises only from 2.97% on day 1 to 5.64% on day 7. This muted growth is immediately superseded by a rapid ascent regime that propels the value to 8.31% on day 14, 12.33% on day 21, 16.33% on day 28, and 19.13% on day 35. Thereafter, the trajectory re-enters a second gradual ascent phase, adding merely 1.99% between days 35 and 42 (21.12%), a further 1.29% by day 49 (22.41%), and finally 1.16% by day 56, when the compression set stabilizes at 23.57%.

The same time–temperature coupling is evident across all isotherms after 7 days the compression set rises from 2.28% at 80 °C to 5.64%, from 2.97% at 100 °C to 6.58%, from 3.34% at 120 °C to 7.59%, and from 5.61% at 140 °C to 10.63%, thereby providing a quantitative basis for extrapolating long-term sealing reliability from short-term thermo-oxidative data.

**Figure 5:**
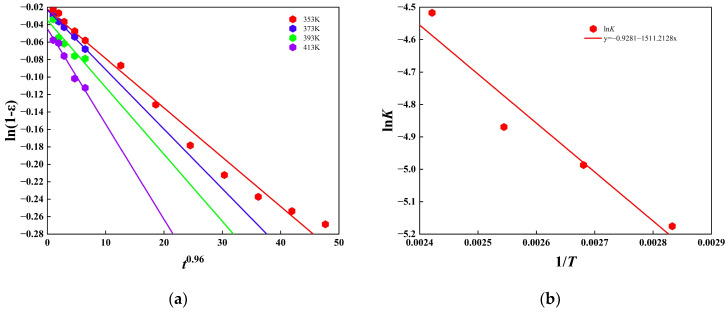
Thermo-oxidative aging lifetime prediction based on Arrhenius kinetics, (**a**) kinetic fitting of ln (1 − ε) and t^0.96^; (**b**) Arrhenius fitting of ln K and 1/T.

The Arrhenius relation ln K = ln A − Ea/RT was invoked to interpret the thermo-oxidative kinetics of phenyl-silicone rubber accelerated aging data collected at 80~140 °C exhibit a strictly linear dependence of ln K on 1/T, validating the assumption of constant activation energy Ea within the degradation window and permitting extrapolation to any lower temperature. Because safety-critical seals are conventionally retired when storage-induced degradation exceeds 10%, whereas an in-service decline of 45% is deemed catastrophic, compression set thresholds of ε = 10% (storage) and ε = 45% (operation) were adopted as failure criteria. Figure 5a demonstrates that plotting the retention variable Y = ln (1 − ε) against t^0.96^ yields a family of straight lines whose slopes deliver the reaction-rate constant K required to reach each critical ε; these K values, in turn, generate the Arrhenius master-curve ln K = −1511.2128/T − 0.9281 shown in Figure 5b. Substitution of the two ε limits into this correlation furnishes a mature life-prediction model at 25 °C, the seal retains functional integrity for 34 days when the 10% storage criterion is invoked, whereas the 45% operational limit extends the notional service life to 286 days, thereby providing quantitative risk-based guidance for both warehouse inventory management and field-replacement schedules.

**Figure 6:**
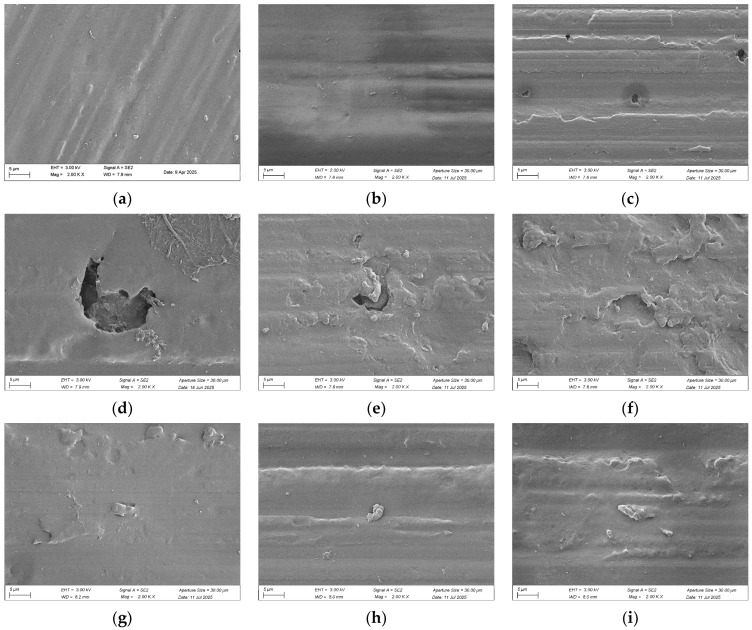
Surface morphology of cylindrical samples after thermo-oxidative aging (2000× magnification): (**a**) Unaged (days 0); (**b**) Days 7; (**c**) Days 14; (**d**) Days 21; (**e**) Days 28; (**f**) Days 35; (**g**) Days 42; (**h**) Days 49; (**i**) Days 56.

Figure 6 assembles a chronological atlas of surface degradation for a cylindrical elastomer subjected to 56 days of thermo-oxidative aging (80 °C, air). The micrographs, recorded at 2000× magnification, chart a morphological trajectory that departs sharply from the classical “smooth-to-rough” corrosion paradigm. Instead, an initially rugged, mold-release topography is systematically planished by oxidative scission and viscous reflow until an almost specular skin emerges.

The unaged sample (Figure 6a) exhibits a relatively rough, featureless surface. After 7 days (Figure 6b), localized pitting initiates across the surface. By day 14 (Figure 6c), pit density has increased substantially, with radial microcracks emanating from cavity edges. At 21 days (Figure 6d), adjacent pits coalesce into shallow, dish-like depressions with rounded perimeters. Following 28 days (Figure 6e), the dimensions of these depressions contract while undulating, and corrugated structures emerge at their margins. After 35 days (Figure 6f), the original depressions become largely effaced as corrugated features proliferate. By day 42 (Figure 6g), all vestiges of cavitation vanish, and corrugations substantially recede. At 49 days (Figure 6h), the surface approaches planar topography, retaining only isolated microparticulate residues. After 56 days (Figure 6i), topographic defects are essentially eliminated, yielding an ordered, terraced morphology. This systematic progression from initial roughness through defect generation to ultimate ordering corroborates the plateau observed in macroscopic compression set curves, indicating that oxidative chain scission and re-crosslinking within the phenyl-silicone rubber network have attained dynamic equilibrium.

**Figure 7:**
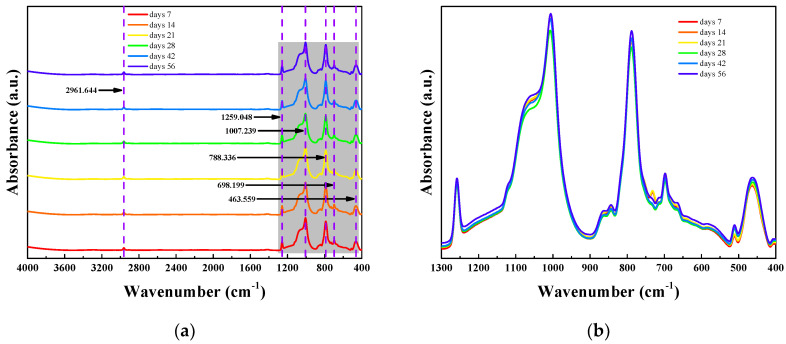
FT-IR absorbance spectra of phenyl-silicone rubber following thermo-oxidative aging: (**a**) spectra of samples aged for 7, 14, 21, 28, 42, and 56 days (full wavenumber range: 400~4000 cm^−1^); (**b**) magnified view of the 400~1300 cm^−1^ region.

FT-IR spectra recorded periodically during 56 days of thermo-oxidative aging (Figure 7) demonstrate that the principal structural moieties of phenyl-silicone rubber-the siloxane backbone and pendant phenyl rings-remain spectroscopically intact. Figure 7a presents full-range spectra (400–4000 cm^−1^) for samples aged 7, 14, 21, 28, 42, and 56 days, showing minimal variation at 2961.6 cm^−1^ (C–H stretching of Si–CH_3_), which confirms the stability of methyl side-chain structures. Figure 7b provides an enlarged view of the fingerprint region (400–1300 cm^−1^), revealing two key phenomena: first, the intensification of peaks at 1259.0 cm^−1^ (Si–O–Si asymmetric stretching) and 1007.3 cm^−1^ (Si–O–Si or Si–OH) indicates thermo-oxidative crosslinking and limited silanol formation; second, the nearly invariant peaks at 788.3 cm^−1^ (Si–CH_3_ bending), 698.2 cm^−1^ (phenyl vibrations), and 463.6 cm^−1^ (Si–O–Si bending) demonstrate that no peak shifting or splitting occurs. This spectral stability confirms the robust thermal-oxidative stability of the phenyl-silicone molecular framework. These subtle spectral changes can be quantified through peak area ratios, serving as sensitive, non-destructive markers for early-stage network oxidation while confirming that the bulk siloxane-phenyl backbone remains structurally intact throughout the aging period.

### 3.3. The Effect of Aging on Sealing Contact Pressure

**Figure 8:**
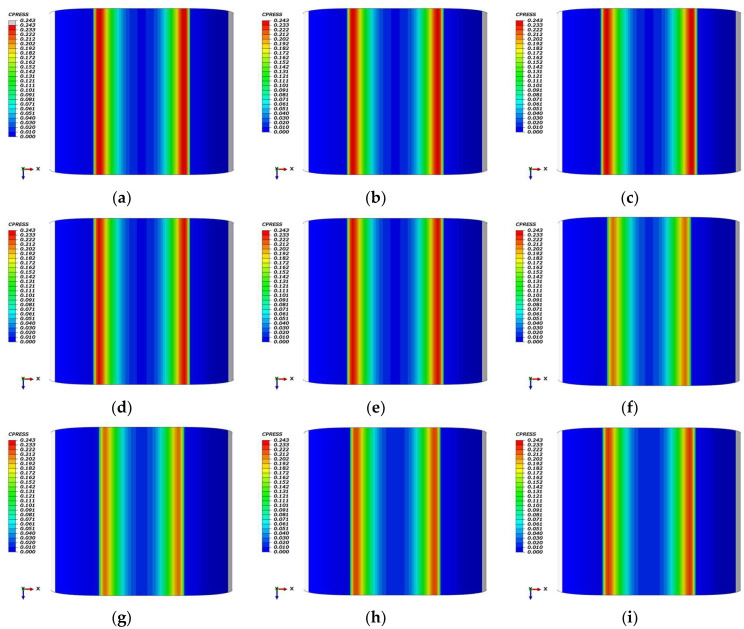
Contact pressure distribution of the Omega-profile seal across temperatures (−70 °C, −55 °C, −25 °C, 0 °C and 25 °C) under different states: (**a**–**e**) unaged; (**f**) unaged at −70 °C with 7.0 mm compression; (**g**) unaged at −55 °C with 7.1 mm compression; (**h**) unaged at −25 °C with 7.5 mm compression; (**i**) unaged at 0 °C with 7.7 mm compression.

Figure 8 illustrates the contact pressure (CPRESS) distributions for an unaged Omega-profile seal across cryogenic-to-ambient temperatures. Figure 8a–e) present baseline distributions without low-temperature contraction effects compensation, showing peak pressures of approximately 0.243 MPa at −70 °C, 0.239 MPa at both −55 °C and −25 °C, and 0.234 MPa at 0 °C and 25 °C, reflecting progressive material softening with increasing temperature. When low-temperature contraction effects are incorporated (Figure 8f–i), the contact pressure response becomes non-monotonic; both −70 °C and −55 °C converge at 0.206 MPa, while −25 °C and 0 °C recover to 0.221 MPa and 0.223 MPa, respectively. This contrasting behavior quantifies the critical influence of low-temperature contraction effects on sealing performance, demonstrating that geometric shrinkage significantly modulates contact pressure distribution across the cryogenic temperature envelope.

**Figure 9:**
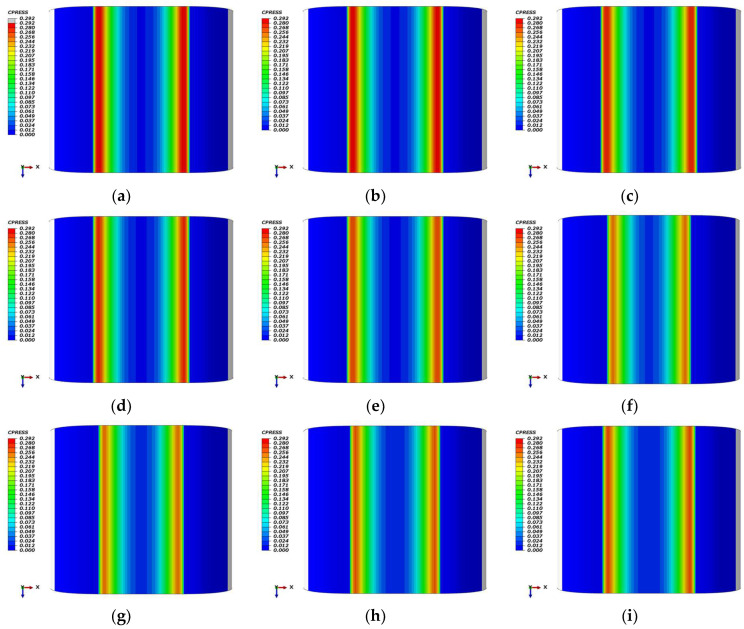
Contact pressure distribution of the Omega-profile seal across temperatures (−70 °C, −55 °C, −25 °C, 0 °C and 25 °C) under different states: (**a**–**e**) 7 days aged; (**f**) 7 days aged at −70 °C with 7.0 mm compression; (**g**) 7 days aged at −55 °C with 7.1 mm compression; (**h**) 7 days aged at −25 °C with 7.5 mm compression; (**i**) 7 days aged at 0 °C with 7.7 mm compression.

Figure 9 illustrates the contact pressure (CPRESS) distributions for an Omega-profile seal following 7 days of thermo-oxidative aging under varying thermal conditions. In the baseline configuration without low-temperature contraction effects compensation, Figure 9a–e demonstrates a progressive reduction in peak contact pressure with increasing temperature, approximately 0.292 MPa at −70 °C, 0.289 MPa at −55 °C, 0.278 MPa at −25 °C, 0.272 MPa at 0 °C, and 0.267 MPa at 25 °C. When low-temperature contraction effects are incorporated (Figure 9f–i), the pressure response becomes non-monotonic, yielding peak values of 0.248 MPa at −70 °C, 0.249 MPa at −55 °C, 0.257 MPa at −25 °C, and 0.260 MPa at 0 °C. This comparative analysis quantifies the critical role of low-temperature contraction effects in modulating sealing performance, revealing that geometric shrinkage partially counteracts the pressure attenuation associated with temperature-dependent material softening across the cryogenic envelope.

**Figure 10:**
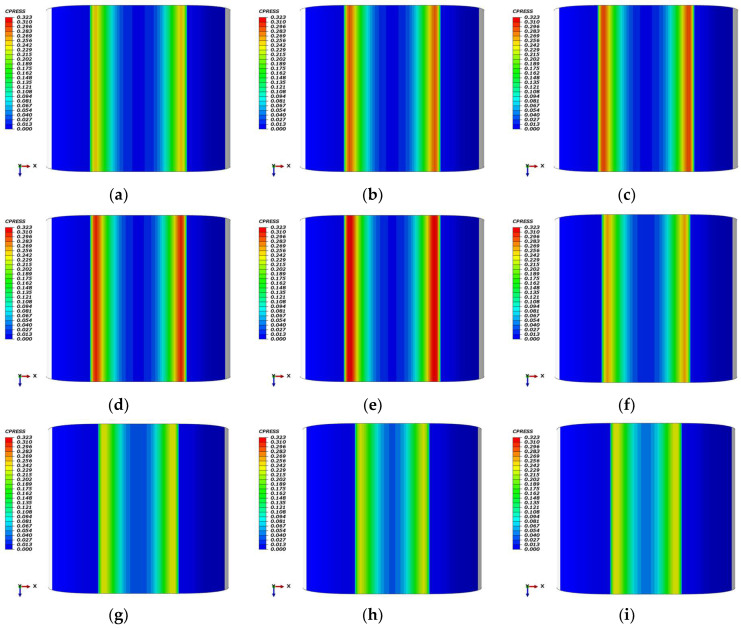
Contact pressure distribution of the Omega-profile seal across aging durations (0, 14, 28, 42 and 56 days): (**a**–**e**) aged; (**f**) days 14 with 7.3 mm compression; (**g**) days 28 with 6.7 mm compression; (**h**) days 42 with 6.3 mm compression; (**i**) days 56 with 6.1 mm compression.

Figure 10 presents the contact pressure (CPRESS) distributions for Omega-profile seals at room temperature following thermo-oxidative aging, contrasting scenarios with and without compression set effects. In the absence of permanent deformation, Figure 10a–e) demonstrates a systematic increase in peak contact pressure with aging duration: the unaged seal (days 0) exhibits approximately 0.246 MPa, which progressively rises to 0.284 MPa at day 14, 0.292 MPa at day 28, 0.300 MPa at day 42, and 0.323 MPa at day 56. Conversely, when accounting for compression set, the cumulative permanent deformation necessitates a reduction in compression displacement to maintain realistic sealing conditions. At day 14, a 0.7 mm displacement reduction (to 7.3 mm) yields a peak pressure of 0.258 MPa (Figure 10f); at day 28, a 1.3 mm reduction (to 6.7 mm) results in 0.241 MPa (Figure 10g); at day 42, a 1.7 mm reduction (to 6.3 mm) produces 0.231 MPa (Figure 10h); and at day 56, a 1.9 mm reduction (to 6.1 mm) generates 0.240 MPa (Figure 10i). This comparative analysis reveals that while thermo-oxidative aging enhances contact pressure through network hardening, the superposition of compression set attenuates this benefit by reducing effective interference, resulting in a 20~25% lower net sealing force compared to the non-set condition at equivalent aging times.

**Figure 11:**
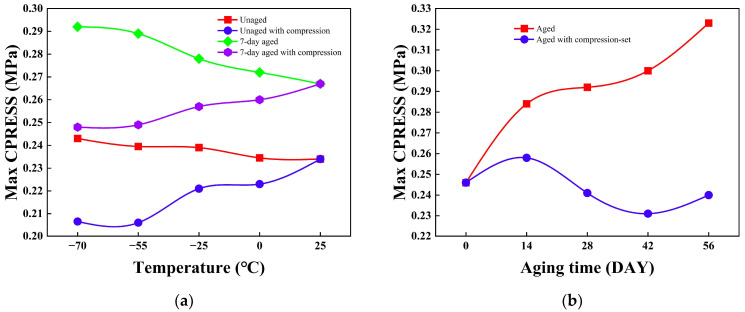
Comparison of maximum contact pressure (Max CPRESS) characteristics of the Omega-profile seal: (**a**) correlation between temperature and Max CPRESS of the Omega-profile seal; (**b**) correlation between aging duration and Max CPRESS of the Omega-profile seal.

Figure 11a systematically compares the maximum contact pressure (Max CPRESS) of unaged and thermo-oxidatively aged Omega-profile seals across the −70 °C to 25 °C temperature spectrum, revealing mechanistically distinct behaviors under two mechanical boundary conditions. When low-temperature contraction is excluded (Figure 11a), Max CPRESS exhibits a monotonic decrease with increasing temperature for both material states, declining by 0.025 MPa (aged) and 0.009 MPa (unaged) over the full range. This inverse temperature dependence originates from the intrinsic thermo-mechanical response of phenyl-silicone rubber: at cryogenic temperatures, suppressed molecular chain mobility induces a “hardening” phenomenon that elevates elastic modulus, while fixed compression displacement and essentially invariant contact area translate this modulus increase into amplified interfacial normal force, thereby raising Max CPRESS. Conversely, as temperature increases toward ambient, enhanced chain mobility reduces modulus and correspondingly diminishes contact pressure. Thermo-oxidative aging further augments Max CPRESS across all temperatures (by 8~21% depending on specific aging conditions), reflecting accelerated chain scission and re-crosslinking that intensify network hardening beyond the baseline thermal effect. When low-temperature contraction is incorporated (Figure 11a), the trend reverses, Max CPRESS increases with temperature, rising by 0.019 MPa (aged) and 0.028 MPa (unaged) from −70 °C to 25 °C. This inversion occurs because cryogenic contraction reduces the effective compression displacement at the contact interface by up to 1.0 mm in aged samples, thereby offsetting the modulus gain from hardening and depressing contact pressure despite the prescribed nominal displacement. Upon returning to ambient temperature, the disappearance of contraction effects enables full interfacial re-engagement, producing higher Max CPRESS values than those observed under cryogenic conditions. Throughout both scenarios, thermo-oxidative aging consistently elevates Max CPRESS by 10~21% relative to the unaged baseline, confirming that oxidative network hardening dominates over the mechanical penalties associated with low-temperature contraction effects.

Figure 11b characterizes the temporal evolution of maximum contact pressure (Max CPRESS) for Omega-profile seals following 56 days of thermo-oxidative aging at room temperature. In the absence of compression set effects, Max CPRESS increases cumulatively by 0.077 MPa from the unaged baseline, reflecting progressive network hardening driven by sustained oxidative penetration. Conversely, when the compression set is incorporated, the trend reverses, Max CPRESS declines by 0.006 MPa over the same period, as irreversible permanent deformation reduces the effective compression displacement and attenuates interfacial normal forces. The aging process exhibits distinct stages at day 14, Max CPRESS rises by 4.878% due to the formation of localized pits and rapid oxidative chain scission/re-crosslinking that sharply elevates elastic modulus; at day 28, it declines by 2.032% as pit coalescence into shallow dish-like depressions occurs and displacement losses from permanent deformation begin to dominate; at day 42, a further 6.098% drop reflects continued deformation override; and by day 56, an additional 2.439% decrease transpires despite the emergence of ordered terraced structures indicating dynamic equilibrium between scission and re-crosslinking. This terminal stage would normally drive modulus recovery and pressure increase, yet the irreversible nature of compression set permanently constrains Max CPRESS below its pristine level.

## 4. Conclusions

By integrating molecular spectroscopy, mechanical characterization, microstructural quantification, and finite-element simulation, this study has established a deterministic, multi-scale framework that links thermo-oxidative aging to the residual life and sealing performance of the phenyl-silicone Omega-profile seals. According to the results above, we have the following points:

1. Early thermo-oxidative aging (≤7 d) stiffens phenyl-silicone rubber (Shore A + 2, 100% modulus elevated across −70 to 25 °C) while simultaneously reducing tensile strength and elongation at break. Microscopy links these bulk changes to a ductile-to-brittle transition: fracture surfaces evolve from rough, dimple-rich profiles to smooth planes perforated by filler-debond pits that deepen into honeycomb porosity-stress concentrators that rationalize the loss in ductility and sealing reliability.
2. Compression set progresses monotonically with time and temperature, saturating at 25% after 56 d at 80 °C. An Arrhenius description (ln K = −1511.2128/T − 0.9281) derived from 80 to 140 °C data predicts 34 d (10% set) and 286 d (45% set) of residual life at 25 °C. SEM reveals a mirror-like oxidative skin coincident with network saturation, corroborating the kinetic model and defining statistically controlled failure thresholds for storage and operational regimes.
3. Finite-element simulations employing experimentally calibrated Neo–Hookean hyper-elastic parameters reveal that contact pressure (CPRESS) in Omega-profile seals consistently peaks at the two lateral necks of the crest. For unaged seals, pressure decreases monotonically from 0.243 MPa at −70 °C to 0.234 MPa at 25 °C; however, inclusion of low-temperature contraction effects reverses this trend, depressing pressures to 0.206 MPa at −70 °C and −55 °C. After 7 days of aging, the Neo–Hookean shear modulus C_10_ rises to 2.27 MPa, elevating CPRESS to 0.267~0.292 MPa. Extended aging (56 days) further increases C_10_ to 2.51 MPa, yielding a cumulative CPRESS gain of 0.077 MPa; yet superposition of compression set reduces effective displacement from 8.0 mm to 6.1 mm, offsetting this hardening benefit and decreasing net pressure by 0.006 MPa (a 20~25% reduction).

## Figures and Tables

**Table 1:** Dimensions of the Type-2 dumbbell sample.

Sample Type	Overall Length	Gauge Length	Width	Thickness
Type-2 dumbbell	75 mm	20.0 ± 0.5 mm	4.0 ± 0.1 mm	2.0 ± 0.2 mm

**Table 2:** Sampling schedule for thermo-oxidative aging.

Sample Type	Temperature/°C	Aging Time/Days											
Dumbbell	100	-	-	-	-	7	-	-	-	-	-	-	-
Cylinder	80	1	2	3	5	7	14	21	28	35	42	49	56
	100	1	2	3	5	7	-	-	-	-	-	-	-
	120	1	2	3	5	7	-	-	-	-	-	-	-
	140	1	2	3	5	7	-	-	-	-	-	-	-

**Table 3:** Environmental chamber temperatures.

Environmental Temperatures/°C				
−70	−55	−25	0	25

**Table 4:** Neo−Hookean hyperelastic parameters C_10_.

Aging Time/Days	C_10_/MPa	Temperature/°C	Days 0	Days 7
0	1.87289134	−70	1.88707728	2.27380648
14	2.20736074	−55	1.85957009	2.24334395
28	2.26921298	−25	1.85630378	2.16330790
42	2.33565305	0	1.81948399	2.11797791
56	2.51338392	25	1.81780786	2.07432047

## Data Availability

Data can be obtained from the authors on request.
